# Medicines, Their Uses and Modes of Administration; Including a Complete Conspectus of the Three British Pharmacopœias, an Account of All the New Remedies, and an Appendix of Formulæ

**Published:** 1844-04

**Authors:** 


Art. VIII.—*Medicines, their Uses and Mode of Administration; including a complete Conspectus of the three British Pharmacopœias, an Account of all the New Remedies, and an Appendix of Formulæ.* By J. Moore Neligan, m.d., Physician to the Jervis-street Hospital, &c. *Dublin,* 1844. 8vo, pp. 432.

This is but an imperfect dispensatory, a critical analysis of which would neither interest nor instruct our readers. In the first place we find a table of contents extending to above twenty-six pages, followed by a most meager description of the numerous substances comprised in it; then comes an imperfect appendix of formulæ, with a posological table: and lastly an index of three or four thousand proper names. An idle pupil, more inclined to trifling amusement than sober study, might perhaps be drilled into habits of perseverance by inflicting upon him such a compilation as this as a task; but we do not understand how the patience of a learned doctor, an hospital physician, and a teacher of therapeutics, could hold out to the last. Still less obvious is it, with the works of Thomson, Paris, Pereira, Merat and De Lens, and Barbier, and various productions under the title of "a complete conspectus" before him, what could induce the author to send the work to press? It is a literary superfluity; and we cannot but think that Dr. Neligan would have done himself and his profession a better service had he illustrated one article of the Materia Medica by original observations and experiments, instead of spending his time on such a compilation. It is, however, but justice to the author to say that his book, although not wanted, will be found very useful to the student. The articles of the materia medica are arranged in it according to their mode of action.

